# Mechanism, prevention and treatment of cognitive impairment caused by high altitude exposure

**DOI:** 10.3389/fphys.2023.1191058

**Published:** 2023-09-04

**Authors:** Xin Chen, Jiexin Zhang, Yuan Lin, Yan Li, Han Wang, Zhanhao Wang, Huawei Liu, Yonghe Hu, Lei Liu

**Affiliations:** ^1^ Department of Clinical Laboratory Medicine, The Third People’s Hospital of Chengdu, The Affiliated Hospital of Southwest Jiaotong University, Chengdu, Sichuan, China; ^2^ Faculty of Chemistry, Chemical Engineering and Life Sciences, Wuhan University of Technology, Wuhan, Hubei, China; ^3^ Sichuan Xincheng Biological Co., LTD., Chengdu, Sichuan, China; ^4^ Department of General Surgery, The 77th Army Hospital, Leshan, Sichuan, China; ^5^ Department of Cardiology, Affiliated Hospital of Southwest Jiaotong University, The Third People’s Hospital of Chengdu, Chengdu, Sichuan, China; ^6^ Faculty of Medicine, Southwest Jiaotong University, Chengdu, Sichuan, China; ^7^ Medical Research Center, The Third People’s Hospital of Chengdu, The Affiliated Hospital of Southwest Jiaotong University, Chengdu, Sichuan, China

**Keywords:** hypobaric hypoxia, cognitive function, mechanism, prevention, therapy

## Abstract

Hypobaric hypoxia (HH) characteristics induce impaired cognitive function, reduced concentration, and memory. In recent years, an increasing number of people have migrated to high-altitude areas for work and study. Headache, sleep disturbance, and cognitive impairment from HH, severely challenges the physical and mental health and affects their quality of life and work efficiency. This review summarizes the manifestations, mechanisms, and preventive and therapeutic methods of HH environment affecting cognitive function and provides theoretical references for exploring and treating high altitude-induced cognitive impairment.

## 1 Introduction

High altitude (HA), which terrestrial altitude above 2,500 m, is one of the most extreme environments on Earth, about 4 hundred million people living there (Leon-Velarde et al., 2005). With increased altitude, the atmospheric pressure decreases and this decrease leads to a decline in the compression of the surrounding air (Palmer, 2010). In brief, as the altitude rises, the density of the air decreases and the partial pressure of oxygen falls (Palmer, 2010). Blood oxygen saturation (SpO_2_) reflects the extent of oxygen bound to hemoglobin in the blood (De Bels et al., 2019). SpO_2_ is a satisfactory indicator of the hypoxic state of the organism. Mean SpO_2_ decreases significantly at high altitudes due to a decrease in oxygen partial pressure in the air (De Bels et al., 2019). Hypoxaemia is defined when SpO_2_ is below 95% (Kim et al., 2013). When SpO_2_ is at 90%–94% mild hypoxemia, 75%–89% moderate hypoxemia, and SpO_2_ less than 75% is diagnosed as severe hypoxemia (Kim et al., 2013). With decreased SpO_2_, the oxygen supply to tissues and organs is reduced, and symptoms such as dyspnea, palpitations, chest tightness, dizziness, confusion, sensory abnormalities, loss of strength, or aches and pains may occur (Henig and Pierson, 2000). The serum of patients with inadequate oxygenation may exhibit low pH and high lactate levels as a consequence of anaerobic metabolism (Henig and Pierson, 2000). Several factors, for instance low pressure, hypoxic conditions, cold temperatures, dry air, and excessive solar radiation, have a persistent impact on human health in HA. Exposure to hypobaric hypoxia (HH) results in many symptoms, such as rapid breathing, faster heart rhythm, nauseous, and headache, which harm the living quality and work efficiency seriously. The brain working is dependent on oxygen, so is very sensitive to HH. Along with altitude rises, HH can trigger cognitive dysfunction of memory, language, visual-space, execution, calculation and understanding judgment (de Aquino Lemos et al., 2012). In severe cases, if hypoxia is not improved continuously, it may lead to HA brain edema. Even after returning to the plain, the cognitive impairment caused by plateau will continue for a long time (Nation et al., 2017).

If the time of HH exposure is longer, the brain damage will be more serious. HH exposure not only affects cognitive physiological and psychological functions, but also may change the structure of the brain (Terraneo and Samaja, 2017). The hippocampus is primarily in charge of memory and learning. The structural integrity of the primary neurons and mitochondrial morphology in the hippocampus would both be substantially compromised by HH (Zhang Z. A. et al., 2022). The main mechanism of HH-induced cognitive dysfunction includes oxidative stress (Li et al., 2017), mitochondrial dysfunction (Li and Wang, 2022), metabolic disorders of nerve cells (Zhang P. et al., 2020), and the increased blood-brain barrier permeability (Zhang et al., 2009). According to above pathogenesis, it is essential to develop prevention and treatment strategies for cognitive dysfunction caused by HH.

Therefore, we aim to summarize the recent research of HH’s effects and mechanism on cognitive function, and provide ideas for the prevention and treatment of cognitive dysfunction caused by HH.

## 2 Cognitive impairments at HAs

The lower atmospheric pressure at altitude causes a decrease in the supply of oxygen in the body when individuals are exposed to high-altitude situations. The brain is highly sensitive to hypoxia, numerous brain functions are impaired (Virués-Ortega et al., 2004; Di Paola et al., 2008).

### 2.1 Effects of acute high-altitude environmental exposure on cognitive performance

From gross inspection, the impairment severity is influenced by the level of altitude gain and higher altitudes have more serious consequences (Yan, 2014). Minimal impairments have been noted at moderate altitudes of 2000–3,000 m, noticeable psychomotor impairments have been seen at 3,000–4,000 m, above 5,000 m, spatial memory was significantly impaired, and impairments in encoding and short-term memory were particularly evident at extremely HAs over 6,000 m (Virués-Ortega et al., 2004; Wilson et al., 2009; Zhang et al., 2011; Bliemsrieder et al., 2022). Furthermore, a study employing the Mood State Inventory, the Clyde Mood Scale, and the Multiple Affective Adjectives Checklist to assess the emotional and cognitive performance of subjects confirmed over 20 years ago that exposure to altitudes above 3,000 m adversely affects individuals’ emotional and cognitive performance. This effect increases with altitude, and test results at 4,700 m showed a significant increase in the severity of this adverse effect compared to 4,200 m (Shukitt-Hale et al., 1998). Cognitive impairment performance is undeniably noticeable within the first one to 2 weeks after individuals have raised to HAs. When exposed to HA for a short time, the human body experiences symptoms such as memory loss, reduced behavior, and poor thinking. Hornbein et al. (1989) reported a slight decrease in language and visual long-term memory along with an increase in errors on the aphasia screening test in mountaineers exposed to altitudes ranging from 5,488 to 8,848 m (Hornbein et al., 1989). One study conducted on an altitude of 3,450 m showed that with only 30 min of acute exposure to hypoxia, impairs individuals’ reaction time, but not complex cognitive performance. And that acute brief exposure to the altitude at which the primary tourist site (about 3,500 m) is located causes substantial executive and memory problems in children (Rimoldi et al., 2016). A systematic meta-regression investigation on the immediate impact of hypoxia on cognition was carried out by McMorris et al. and they found that blood oxygen partial pressure is a critical predictor of cognitive capacity (independent of whether the exposure was to low-pressure or normobaric hypoxic conditions) and that studies testing both executive abilities such as transfer of working memory set, monitoring, suppression and planning, and non-executive abilities including short-term memory, perceptiveness, and attention, were adversely affected by hypoxia (McMorris et al., 2017). Another systematic review with meta-analysis confirmed the impact of hypoxia on cognition (independent of whether the exposure was to low-pressure or normobaric hypoxic conditions), and researchers observed a selective effect: information processing seemed to be improved (especially in women), but executive ability and memory seemed to be impaired (Jung et al., 2020). Changes in the speed, length, and profile of exposure at altitude, method of ascent, study population, cognitive measures used, and time spent conducting tests at altitude can explain inconsistencies and impede conclusions about the effects of altitude on cognition (Li et al., 2000; Davranche et al., 2016; Loprinzi et al., 2019b; De Bels et al., 2019).

### 2.2 Effects of long-term high-altitude exposure on cognitive performance

Long-term exposure to high-altitude hypoxia impairs cognitive function manifested in a decrease in inhibitory control, attention and memory (Li and Wang, 2022). Cognitive impairments have been noted in long-term high-altitude populations, and the severity of the hypobaric hypoxic effect may rely on the period of living at HAs. A study simulating environments ranging from sea level to 8,848 m altitude observed that individuals chronically exposed to HAs continue to exhibit a severe deficit of color recognition capability (Bouquet et al., 2000). Event-related potentials (ERPs) have been reported to be affected by hypoxia, which in turn causes cognitive impairment. By exploring the effects of chronic HH on ERPs at two different altitudes, 3,200 and 4,300 m, the study found that HH causes a slowing of signal processing at 4,300 m and longer ERPs wave latency at higher altitudes (Singh et al., 2004). As the duration of exposure prolongs, there is an initial occurrence of high-altitude hypoxic acclimatization, and the cognitive function of the individual recovers slightly, but it is still hard to fully reach the level of subjects in the plain (Zhu and Fan, 2017). Moreover, the color reaction time and short-term visual memory of individuals showed a gradual decline with the extension of the duration of stay in the HA (Zhu and Fan, 2017). The duration of the high-altitude hypoxia adaptation period increases with altitude. The higher the altitudes, the more severe impact of hypoxia on physiological effects, and the more pronounced the decline in human attention and work capacity (Thakur et al., 2011; Li and Wang, 2022). In general, cognitive function declines first, then rises moderately, and finally decreases with the duration of altitude exposure increases, remaining in a state of impairment (An et al., 2017). People exposed to a high-altitude environment for a longer time have more apparent damage to the reaction time, depth perception, operating-dexterity, and attention (Bao et al., 2014). Notably, long-term exposure to an altitude of 3,450 m caused severe executive and memory deficits in children who lived permanently at HAs and were expected to impair their learning capacities (Rimoldi et al., 2016).

Functional magnetic resonance imaging (fMRI), and diffusion tensor imaging (DTI), voxel-mirrored homotopic connectivity (VMHC) and event related potentials (ERP) were employed to further reveal the brain function basis for cognitive function changes (Zhang et al., 2013a; Chen et al., 2016). The prolonged hypoxic exposure causes anatomical changes throughout the brain, with gray matter throughout the brain atrophying (Zhang et al., 2013a). Long-term hypoxia impairs attention perform, executive function, memory, and other cognitive functions in lowlanders at HAs by affecting ERP components in the parietal occipital lobe, prefrontal lobe, anterior cingulate cortex, temporal lobe, and other brain regions (Zhang et al., 2013a; Wang et al., 2014; Zhang D. et al., 2018; Ma H. L. et al., 2020). Therefore, as the time of high-altitude environmental exposure increased, the attention, information processing rate, spatial cognitive ability, and executive function of the subjects all showed different degrees of impairment. The longer the exposure time, the more serious the damage of cognitive function.

## 3 Integrative physiology of human cognitive responses to high-altitude

The environment at HAs may directly affects individuals’ cognitive functions such as short-term memory, judgment, and attention span, which may pose a threat to an individual’s physical and mental health (de Aquino Lemos et al., 2012; Wang et al., 2013). Previous research has found that in the large mining industry in the Chilean mountains or at the Atacama Large Millimeter/Submillimeter Array (ALMA) scientific observatory (5,050 m), HH impairs the cognitive performance of high-altitude workers and may lead to higher error rates (Hornbein et al., 1989; Davranche et al., 2016). On this basis, Matiram et al. investigated the effects of acute, habitual, and repeated exposure to very HAs on individual cognitive function. They used SpO_2_ and AMS scores to explain cognitive variations during high-altitude exposure and acclimation, emphasizing the importance of the acclimation period for recovery of cognitive function under high-altitude exposure. It was also noted that the improvement in cognitive function during the habituation period of initial exposure did not carry over to the period of repeated exposure (Pun et al., 2018). The first meta-analysis of high-altitude mountaineers was conducted to evaluate the effects of high-altitude mountaineering on the cognitive function of mountaineers in four perspectives: motor speed, memory function, executive function, and language function (Li et al., 2023). This study found that high-altitude mountaineering had significant adverse effects on motor speed and verbal working memories, but no significant adverse effects on executive function and verbal function in mountaineers. It may be attributed to the short-term exposure to high-altitude mountaineering being insufficient, and further research is needed for prolonged high-altitude mountaineering and exploration of executive and verbal functions. As the hippocampus and other areas within the limbic system of the brain are very sensitive to hypoxia, the exponentially decreasing of partial oxygen pressure will lead to brain symptoms such as headache, vomiting, nausea, impaired coordination, and neurocognitive dysfunction in humans (Hornbein, 2001; Bärtsch and Swenson, 2013). Brain structural and vascular system changes are thought to underlie cognitive deficits such as decreased attention, executive dysfunction, reduced information processing, and memory dysfunction induced by altitude hypoxemia (Hornbein, 2001). In addition, early studies have found that HH can affect neuropsychological performance and can impair an individual’s mental and physical health and general wellbeing (Gerard et al., 2000). A study of the effects of HA in ALMA (5,050 m) on different cognitive domains in year-round shift workers, by assessing processing speed, attention, and executive ability, found that regular rotation of work cycles could be adapted to high-altitude hypoxic environments to some extent. And preferably through oxygen enrichment to improve blood oxygen levels to optimize the cognitive performance of ALMA workers and reduce altitude-related health risks (Pun et al., 2019). Further, Fernando et al. assessed pulse oximetry, arterial systolic and diastolic blood pressure, and neuropsychological tests in workers exposed to the ALMA radio telescope at 5,050 m altitude, and discovered that simulating 28% ambient oxygen enrichment using a removable oxygen module system significantly improved cardiorespiratory responses and psychoneurological function (Moraga et al., 2018). For high-altitude staff, the labor risks associated with high-altitude hypoxia can be reduced by improving blood oxygen concentrations through shift work, acclimatization, and the use of mobile modular oxygen systems.

High-altitude exposure induces adverse effects on cognitive performance and mood, potentially due to poor sleep quality (de Aquino Lemos et al., 2012; Morrison et al., 2017). High-altitude travelers experience frequent arousals and low slow-wave sleep duration, which can impair attention, memory, time to exhibit complex responses, and perceptual-motor function, resulting in increased irritability and depression (Dewald et al., 2010; Rosenzweig et al., 2015). HH triggers erythrocytosis, and research has found that soldiers with erythrocytosis stationed on the Tibetan plateau have poorer sleep quality than healthy soldiers, which negatively affects cognitive function. And impaired sleep quality can predict decreased cognitive function, typically in linguistic and short-term memory (Kong et al., 2011). Adaptive servo-ventilation (ASV) can improve sleep quality by stabilizing breathing and preventing central apnea without supplemental oxygen compared to supplemental oxygen sleep therapy. A study of high-altitude travelers found that using either supplemental oxygen or ASV during sleep reduced fatigue and improved certain aspects of cognitive function, including executive control, sustained attention, and risk inhibition (Heinrich et al., 2019). As well, high-altitude HH can affect sleep quality and reduce a person’s alertness. Acute exposure to HH decreases the psychomotor vigilance response rate (PVT-RS). A clinical trial conducted at the ALMA Observatory in Chile at 5,050 m found that exposure to HH for more than 6 days restored PVT-RS in humans. However, total sleep time did not improve during acute and subacute exposure (Pun et al., 2018).

Diseases associated with altitude include acute high-altitude illness and potentially fatal high-altitude cerebral edema and high-altitude pulmonary edema and so on also has effects on cognition. Acute high-altitude illness manifests with headache, vomiting, and sleep disturbance, and can be alleviated to some extent by the practice of clothing and supplemental oxygen (Goldfarb-Rumyantzev and Alper, 2014). A study of climbers on Mount Monterosa (4,554 m), which correlated cognitive performance with other measures of brain status by standardizing acute plateau sickness scores and measuring serum concentrations of the central neuro-specific protein S100B, found that cognitive decline was associated with acute plateau sickness (Bjursten et al., 2010). High-altitude cerebral edema and high-altitude pulmonary edema manifest as fatal symptoms of brain dysfunction, including persistent headache, ataxia, and confusion, often accompanied by hypoxemia, respiratory distress, and impaired motor ability (Goldfarb-Rumyantzev and Alper, 2014). Hypoxemia can lead to brain damage, neurological and cognitive dysfunction, and even death. Research has attributed altitude pulmonary edema to hypoxia-induced pulmonary vasoconstriction causing increased pulmonary artery pressure and noncardiogenic pulmonary edema (Maggiorini, 2010). While other studies show that compared healthy individuals and patients with high-altitude pulmonary hypertension found that brain tissue oxygenation in patients with high-altitude pulmonary hypertension at 3,250 m above sea level was similar to that of healthy individuals at 760 m altitude (Furian et al., 2015). This requires of course further verification at very HAs.

## 4 Mechanism of high-altitude hypoxia environment affecting cognitive function

Changes in physiological mechanisms are what caused by HH to have an impact on cognitive performance. The occurrence of cognitive impairment under high-altitude exposure may be closely related to oxidative stress response, neurotransmitters, neuronal cell injury, involvement of hypoxia inducible factor, and inflammatory response. We summarize the relationship between HA exposure and cognitive function at four levels: neural mechanisms, stress mechanisms, cellular mechanisms, and molecular mechanisms.

### 4.1 Neural mechanism

Oxygen supplies the brain with energy and is associated with the rapid development of inter-neuronal connectivity and synaptic activity (Seymour et al., 2016). Reduced SpO_2_ is associated with the brainstem and the cerebral cortex. The brainstem is the area controlling basic physiological functions such as respiration and heart rate (Mehta et al., 2016), and the cerebral cortex is primarily responsible for sensory, cognition, and motility (Bayraktar et al., 2020). It has been discovered that the insular cortex on the cerebral cortex, which is the sensory center of the viscera, is expected to be affected by SpO_2_ variations (Zhang and Zhang, 2022). Inhalational hypoxia stimulates an increase in cerebral blood flow (CBF) to maintain oxygen delivery to the brain (Harris et al., 2013). The response of CBF to hypoxia is dynamic, evolving with the duration and degree of hypoxic exposure (Wilson et al., 2011). Zhang et al. and Kottke et al. found that brain structures may be affected by variations in CBF induced by alterations in the state of oxygenation, by acquiring brain images of highland explorers (Zhang et al., 2012; Kottke et al., 2015). The study by Naftali et al. revealed that cerebral white matter hyper-signaling associated with cerebral ischemia and hypoxia was associated with cerebrovascular risk factors and mild cognitive dysfunction (Raz et al., 2007). People exposed to HA have been observed to suffer from cerebral vasodilatation, increased CBF, and accompanying intracranial fluid transfer from extracellular to intracellular, with fatal consequences that culminate in the development of HACE or even coma (Hackett and Roach, 2004; Wu et al., 2006). Malignant changes in brain structures manifest cognitive dysfunctions such as confusion, delirium, altered consciousness, and behavioral abnormalities (Turner et al., 2021). Therefore, exposure to HH can lead to structural changes in the brain and neurological deficits, with pathological changes largely dependent on the duration of hypoxia and altitude (Wang et al., 2022). The hippocampus, which is involved in long-term and visual-spatial memory, is the most sensitive cerebral region for hypoxia (Eichenbaum, 2000). When rats were exposed to HH, neuronal fixation, neuronal degeneration, and apoptosis were noted in both CA1 and CA3 regions of hippocampal pyramidal neurons, which may be the major cause of impaired neural circuit stability and memory impairment in hippocampus (Maiti et al., 2007). The striatum and neocortex are also fragile constructions that play a crucial role in sensory, concentration, and long-term memory (Eichenbaum, 2000; Goldfarb et al., 2016). MR imaging revealed that the thickness of the bilateral cerebral regions, the right front cingulate girdle, the bilateral prefrontal cortices, the left anterior ventral cortex, and the right linguistic cortex were significantly increased with high-altitude HH exposure. The thickness of the corresponding regions of the brain mentioned above decreased significantly with prolonged altitude exposure (Zhang et al., 2010; Zhang et al., 2013a). The findings above suggest that when exposed to hypoxia, the brain appears to have localized cerebral vascular hyperplasia and increased cortical thickness in local brain regions to compensate for insufficient blood oxygen concentration. With extended exposure, the entire brain grey matter shows a tendency to atrophy and exhibits non-specific damage characteristics (Zhang et al., 2010). Previous diffusion tensor imaging (DTI) of the entire brain white matter analysis confirmed these results. The fractional anisotropy (FA) of brain tissue was reduced in individuals entering the plateau in the bilateral superior and inferior longitudinal tracts, corpus callosum, corona radiata, posterior cingulate gyrus and corticospinal tract fractions, corresponding to an increase in FA with prolonged exposure to HA (Zhang et al., 2010; Zhang et al., 2012). In addition, it was found that reduced grey matter volume in the parahippocampal and middle frontal gyrus was positively correlated with changes in lung volume, and changes in postcentral gyrus cortex thickness were associated with reaction time and memory in the high-altitude exposed population (Zhang et al., 2012). Curiously, it has been proposed that changes in total brain white matter and brain parenchyma volume significantly correlate with SpO_2_ when the organism is in static hypoxia, however, there is no correlation between brain volume changes and SpO_2_ when it is in motion hypoxia (Rupp et al., 2014). In general, prolonged exposure to high-altitude HH induces conformational changes in the brain, which may be the anatomical basis for cognitive impairment.

### 4.2 Stress mechanism

When the body is under attack, it produces stress, a variety of physiological and psychological reactions, to maintain equilibrium (Li and Wang, 2022). Oxidative stress occurs as a response to an imbalance in the overproduced free radicals in the body and the antioxidant defenses of the cells themselves (Erukainure et al., 2018). Hypoxia-induced oxidative stress is one of the major contributors to cerebral damage and cognitive impairment. Exposure to HH leads to high-altitude hypoxic stress, through physiological compensatory mechanisms such as raised ventilation frequency, enhanced heart pulse rate, vasodilation, increased blood cells, and improved Blood flow to the brain to maintain homeostasis in the body (Quillinan et al., 2016). Under these situations, the production of reactive oxygen species (ROS) and reactive nitrogen species (RNS) increases (Kalyanaraman, 2013). Rats exposed to HH have been observed to develop oxidative stress associated with memory impairment. The accumulation of large amounts of ROS and RNS in the body directly impairs the basement membrane of the blood-brain barrier (BBB), leading to vasogenic edema, and causing disruptions in the oxidative and antioxidant systems in the brain, resulting in brain damage and cognitive impairment. In addition, free radicals can also inhibit synaptic transmission, cell proliferation, and neuronal differentiation through the activation of protein phosphatase 2A (PP2A), all of which are essential factors contributing to cognitive deficits (Chen et al., 2014; Bowser et al., 2018).

Studies found that the accumulation of free radicals can also induce neuroinflammation by activating microglia and astrocytes, causing glial cells to produce excessive amounts of pro-inflammatory and inflammatory cytokines such as interleukin-1β (IL-1β), IL-6, IL-1α, tumor necrosis factor-α (TNF-α), interferon-gamma (IFN-γ), as well as glutamate (Jellema et al., 2013). Production of TNF-α may impair the BBB, promote BBB transport proteins dysregulation, disrupt the extracellular matrix and neurovascular, and then lead to leukocyte migration and glial cell activation. Cerebral disturbances may eventually affect synaptic plasticity and contribute to cognitive impairment (Rosenberg, 2017). Mice exposed to HH exhibit hippocampal-mediated memory deficits accompanied by exceptional magnetic resonance (MR) imaging of the brain, which is consistent with neurovascular alterations, systemic inflammation, and white matter remodeling (Shi et al., 2012). This study indicates that the vascular remodeling and inflammation induced by high-altitude exposure serve for the induction of cognitive deficits (Shi et al., 2012). In addition, the occurrence of high-altitude stress also reduces cellular mitochondrial biosynthesis through the downregulation of peroxisome proliferator-activated receptor-γ coactivator-1α (PG1α) expression, which leads to rapid morphological damage to cellular mitochondria, cell swelling, and necrosis (Zheng et al., 2019). The chronic imbalance in antioxidant systems induced by long-term residence at HAs induces systemic nitrosative inflammatory stress and accelerates the development of cognitive dysfunction in patients with chronic altitude sickness (Bailey et al., 2019). The oxidative stress from high-altitude exposure may also raise the risk of depression, bipolar disorder, and committing suicide in individuals (Hwang et al., 2019).

### 4.3 Cellular mechanism

The most common symptoms of altitude sickness, including dizziness, headache, shortness of breath, and fatigue, are experienced when first entering HAs (Ding et al., 2018). These are connected to the characteristic HH environment of the plateau. During acute hypoxia, the body undergoes a compensatory response, initiating systemic cardiopulmonary reflexes, leading to vasodilation and hyperventilation (Sharp and Bernaudin, 2004). Prolonged hypoxia induces an increase in oxygen-transporting red blood cells, an increment in blood viscosity, and a clinical diagnosis of high-altitude polycythemia (HAPC), whose negative effects on individual cognitive function have been observed (Li and Wang, 2022). On the one hand, HAPC induces cumulative alternations in cerebral and local delicate organization structure and function, and impaired cognition occurs. Morphometric findings reveal that patients with HAPC have increased grey matter volumes in the right lingual gyrus, post-banding gyrus, bilateral parietal gyrus of the hippocampus, and left inferior temporal gyrus compared to normal, and decreased volumes in the left anterior cingulate gyrus compared to normal, representing potential risks for impaired visual and cognitive function (Li and Wang, 2022). On the other hand, the sensation of brain pain and shortness of breath brought on by HAPC can affect the quality of sleep of individuals, leading to a deterioration in attention, mental flexibility, and memory (Pelamatti et al., 2003; Li and Wang, 2022).

Additionally, at the subcellular level, mitochondria play a crucial role in the overall functional changes induced by hypoxia (McKenna et al., 2020). With high-altitude HH exposure, glucose metabolism in the brain converts to anaerobic glycolysis, production of cellular ATP is drastically reduced and pyruvate production in the brain increases, promoting lactate accumulation and potentially causing neurological damage (Erecińska and Silver, 1994). In this situation, an increase in mitochondrial bulk density and cristae abundance, with cristae fragmentation and distinctive beehive-like structures to resist oxidative stress (Perkins et al., 2012). At the same time, brain adenosine concentrations increase rapidly following hypoxia, activating adenosine A1A receptors to lead to the inhibition of hippocampal salience transmission (Kawamura et al., 2019).

### 4.4 Molecular mechanism

During the phase of hypoxia-induced compensatory response of the organism, the hypoxia-inducible factor (HIF) erythropoietin (EPO) pathway is activated to obtain more oxygen, promoting the secretion of large amounts of EPO by the liver and kidneys, an increase in hemoglobin concentration and erythrocyte pressure-volume, and consequently an increase in red blood cells (Li and Wang, 2022). HIF-1α is a heterodimeric protein that produces transcription factors and consists of β subunit (HIF-β) and the alpha subunit (HIF-α) (Kaelin and Ratcliffe, 2008). During hypoxia, the HIF-α subunit accumulates and undergoes nuclear translocation, inducing the inactivation of proline-4-hydroxylase (PHD) and promoting activation of NF-κB (Pan et al., 2021). NF-κB is pivotally important in the inflammatory response and causes neuronal damage and reactive gliosis, leading to impaired learning and memory deficits (Angelo et al., 2014; Bowser et al., 2018). Besides, HIF-α can be activated by free radicals through the MAPK/P13K/Akt signaling pathway (Movafagh et al., 2015). Studies have shown that HIF-1α could induce brain injuries by promoting the activation of neuronal autophagy and triggering HAPC, leading to cumulative structural and functional changes in the brain (Niu et al., 2018). Furthermore, HIF-1α can induce neuronal apoptosis by upregulating apoptotic factors such as caspase-3, Bax, Bcl-2, and activation of the HIF-1α/heme oxygenase-1 signaling pathway, leading to hippocampal and cortical atrophy and ventricular enlargement (Chen et al., 2013).

Recent findings suggest that induction of cold-inducible RNA-binding protein (Cirbp) overexpression in the hippocampal region attenuates HH exposure-induced hippocampal dendritic spine injury and cognitive impairment in mice (Zhou et al., 2021). In addition, overexpression of vascular endothelial growth factor (VEGF) and activation of the Wnt/βcatenin pathway, c-Fos, and NGFI-A pathways may trigger vascular neovascularization in response to chronically hypoxic conditions (Rybnikova et al., 2009; Tsai et al., 2013; Sun et al., 2020).

## 5 Prevention

At present, the preventive measures for altitude cognitive dysfunction mainly include plateau acclimatization and nutritional supplement. Plateau acclimatization is a process in which the human body produces physiological changes to adapt to the plateau hypoxia environment after entering the plateau from the plain for a period of time. Before entering the plateau, we can improve the physical fitness, enhance the tolerance to hypoxia, or use adaptive service ventilation system to reduce the cognitive damage of HH. The methods of promoting acclimatization mainly include controlling the ascent rate, hypoxic preconditioning, adaptive exercise, Vitamin and dietary supplement and others.

### 5.1 Controlling the ascent rate

One of the most effective way for preventing altitude cognitive dysfunction is plateau acclimatization through controlling the ascent rate (Johnson and Luks, 2016). For this, some institutions put forward suggestions. The Himalayan Rescue Association recommends that controlling the ascending to no more than 300 m/day and with a rest day for each additional 600–900 m could effectively adapt to plateau. The Wilderness Medical Society suggested that the ascent limit was controlled no more than 500 m/day and with a rest day for every 3–4 days could effectively alleviate the discomfort caused by elevation rise (Zafren, 2014). Guo et al. showed that when exposed to a high-altitude at 4,400 m, 4-day short-term high-altitude pre-exposure at 3,700 m has a better effect in improving human neurobehavioral parameters by comparing to 3-month long-term exposure, such as mood states, cognitive performance and acute mountain sickness (Guo et al., 2016). Nisha et al. also found that the group of aviation personnel who had chronic intermittent exposure to hypobaric hypoxic environment, did not have any significant decrease in cognitive function, namely, attention, decision-making and problem solving compared to controls during a working memory task (Nisha et al., 2020).

### 5.2 Hypoxic preconditioning

Hypoxic preconditioning is a method for the body to improve hypoxia tolerance through its self-protection mechanism. Generally, intermittent hypoxia in the low pressure chamber is used to make the body obtain tolerance to secondary hypoxia injury through one or more hypoxia stimuli, so as to improve exercise tolerance and central fatigue under severe hypoxia conditions (Wang et al., 2016). Beidleman et al. verified that 3 weeks of intermittent altitude exposures (the simulated altitude of 4300 m plateau was 4 h a day, 5 days a week) provide an effective alternative to chronic altitude residence, which could increase the resting ventilation and reduce the incidence or severity of acute mountain sickness (Beidleman et al., 2004). Katayama et al. indicated that in the hypoxic tent with 12.3% oxygen content, 1 h or 3 h a day for 7 consecutive days can significantly enhance the hypoxic ventilatory response of the subjects (Katayama et al., 2009). In addition, exposure to intermittent hypoxia was beneficial for hypoxic preconditioning. Intermittent hypoxia could prevent cell damage and benefit for therapy. Intermittent HH exerted neuroprotection against acute severe hypoxia induced oxidative injury through preventing oxidative stress and inhibiting the apoptotic cascade, which was associated with NF-κB downregulation and erythropoietin upregulation (Coimbra-Costa et al., 2021). However, another study reported that when rats were exposed to a simulated high-altitude exposure, hyperbaric oxygen preconditioning could prevent the occurrence of cerebral and pulmonary edema (Lin et al., 2012). In addition, a cohort study in young migrants in Tibet indicated that single session of hyperbaric oxygen intervention significantly improved the orienting function of attention, and there was a strong association between alerting function and conflict function after the end of intervention, suggesting the change of the overall performance of attention function (Bu et al., 2021).

### 5.3 Adaptive exercise

Adaptive exercise refers to long distance running, weight bearing stretching and other training before entering the plateau to improve the adaptability under hypoxic environment. It has been confirmed by many studies that adaptive exercise can improve cognitive performance and prevent cognitive dysfunction (Komiyama et al., 2017; Loprinzi et al., 2019a). According to a recent review, various characteristics exercises may regulate the relationship between exercise and cognitive performance under hypoxia (Jung et al., 2020). Exercise was shown to improve neural activity, so it could improve the cerebral vasculature and cognitive functions (Ohline and Abraham, 2019). Koester et al. also suggested that exposure to exercise is profit for recovering the cognitive function impaired by HA. In addition, the VEGF signaling is important on maintaining neurons, neovascularization and neurogenesis at HA (Koester-Hegmann et al., 2018).

As we all know, Reinhold Messner is a famous climber and mountaineer who climbed to the summit of the world’s top mountain, Mount Chomolungma, without oxygen in 1978. There are several possible reasons for his ability to complete an anoxic ascent in a relatively short period. Firstly, Reinhold Messner possesses outstanding physical and physiological qualities. Long-term altitude training and mountaineering experiences have allowed him to gradually adapt to high-altitude environments, and his body can more effectively utilize limited oxygen resources and quickly adapt to reduced oxygen. Most studies have found that training at moderate altitudes (2000–3,000 m) improves adaptability and endurance at high altitudes and improves athletes’ performance (Flaherty et al., 2016; Khodaee et al., 2016). Highland hypoxia exposure is a key environmental stressor that initiates critical physiological adaptations in athletes. HIF is a critical factor for these physiological adaptations, functioning as a transcription factor and a major regulator of oxygen homeostasis (Caro, 2001; Semenza, 2004). Under normoxic conditions, HIF1 is rapidly degraded by the ubiquitin-proteasome pathway and is undetectable (Wilber et al., 2007). Its degradation significantly slows down during hypoxic exposure, resulting in increased half-life and transcriptional activation of target genes encoding erythropoietin (EPO) and other molecules (Mazzeo, 2008). In a nutshell, plateau hypoxia training improves tissue oxygenation and limits hypoxic injury. Secondly, as an experienced mountaineer, Reinhold Messner has mastered the techniques and tactics of high-altitude mountaineering. He understands how to rationalize climbing routes and select appropriate time and weather conditions to minimize the time consumption of the climbing process. Thirdly, Reinhold Messner has a strong inner motivation and determination to challenge the extreme and overcome the ego. This psychological quality enables him to push forward steadfastly under grueling conditions, undaunted by difficulties and risks.

### 5.4 Vitamin and dietary supplement

The potential role of B-vitamins in maintaining cognitive function was well known (Morris, 2012). Yu et al. reported that supplementation of vitamin B6/B12/folate and choline could observably improve the memory deficits which induced by hypoxia, and the supplementation of B-vitamins and choline could also decrease the concentration of homocysteine in serum and tau hyperphosphorylation at multiple AD-related sites by upregulation of Ser9-phosphorylated GSK-3β (Yu et al., 2016). These findings provide new ideas for combine of B-vitamins and choline to protect cognitive function against hypoxia (Yu et al., 2016).

Sugar-sweetened beverages (SSB), a class of very popular non-alcoholic beverages throughout the world, are characterized by high added sugar content, especially fructose-containing sugar (Hu et al., 2019). Zhang et al. suggested that SSB consumption was associated with poorer executive function in Chinese Tibetan adolescents. SSB consumption should be controlled for healthy brain development of Chinese Tibetan adolescents (Zhang F. et al., 2022).

Ketogenic diet (KD), a high-fat with low-carbohydrate diet, has been reported as an effective means on cognition and behavior in various neurological disorder (Hallbook et al., 2012). In animal experiment, Hallbook et al. demonstrated that KD treatment could not only enhance the spatial learning and memory, but also improve the spatial memory impairment induced by HH (Zhao et al., 2017).

### 5.5 Others

In addition, mood also has an impact on cognitive function after HH exposure. Karinen et al. investigated the cognitive function changes of 9 climbers who climbed Mount Everest and found that the climbers with good subjective enthusiasm and strong psychological quality show a more stable emotional state and a better level of physical vitality (Karinen and Tuomisto, 2017). Another interesting study shows that advance meditation in the HAs was helpful for improving biochemical and neuro-cognitive (Bhanushali et al., 2020). Patrician et al. indicates that a simple noninvasive and portable dead space mask resulted in reductions (49%) in apnea-hypopnea index, and reduced headache severity and aspects of cognitive decline at hypobaric hypoxia (Patrician et al., 2019).

## 6 Therapy

If the cognitive dysfunction caused by HH cannot be alleviated by prevention, it could be solved by some intervention measures. The treatment strategies mainly include Physiotherapy and medical treatment.

### 6.1 Physiotherapy

Electrical stimulation of cerebellar fastigial nucleus is a useful technique for promoting neurological functional recovery and reducing infarct volume against cerebral ischemia (Liu et al., 2012; Mandel et al., 2012). A recent study pointed out that fastigial nucleus stimulation (FNS) improved cognitive function by reducing the prolonged latencies of event related potentials and decreasing the average velocity of brain arteries at 4,000 m altitude. FNS may be a potential and effective method for the treatment of cognitive dysfunction at HAs (Hu et al., 2017). Transcranial direct current stimulation (TDCS) is a neural regulation technology and can upregulate or downregulate the excitability of specific brain regions, significantly improve cognitive ability (Brunoni and Vanderhasselt, 2014; Coffman et al., 2014), which is generally used to treat nervous system diseases, such as severe depression, Parkinson’s disease, chronic pain, etc. (Nelson et al., 2014; Douglas et al., 2015). In recent years, some reports showed that TDCS can increase local cerebral oxygen saturation, improve fatigue, enhance energy, and improve some plateau brain cognitive ability and plateau mental work ability to a certain extent (Dalong et al., 2022).

Enhancing the oxygen carrying of the body can improve the cognitive dysfunction caused by HA. Another study on remote ischemic preconditioning (RIPC) indicates that after 1 week of Remote ischemic conditioning (RIC) treatment, the attention early warning function of subjects exposed to HA was significantly improved (Li et al., 2020). Wu et al. also found that the RIPC treatment improved spatial memory and sleep quality in subjects exposed to acute hypoxic exposure and this may lead to improved performance at HAs (Wu et al., 2023). The mechanism of RIPC protects cognitive function is unknown, but it was still a promising treatment strategy for cognitive dysfunction by HH. Wu et al. found that the heat shocks protein-70 mediated hyperbaric oxygen therapy could ameliorate spatial memory dysfunction and passive avoidance learning of the rat after high-altitude exposure (Wu et al., 2018). Heinrich et al. demonstrates that ASV could improve the sleep quality of subjects at night, reduce the sense of fatigue, alleviate cognitive dysfunction, improve willpower and sustained attention (Heinrich et al., 2019).

Oxygen enrichment is increasingly recognized as an effective approach to reduce the equivalent altitude, which can raise the oxygen concentration to mimic higher oxygen partial pressure of a lower altitude. West JB proposed that every 1% increase in oxygen concentration in the oxygen-enriched chamber equates to a reduction in elevation of approximately 300 m (West, 1999). Routinely used clinical concentrations of oxygen therapy are 50% or 30%, whereas Dong Yan et al. reported a superior rescue of patients with hemorrhagic shock at HAs employing high-concentration oxygen therapy of 80% (Yan et al., 2004). Additionally, in contrast to previous high-flow oxygen therapy, Pattinson et al. successfully treated a patient with HACE utilizing low-flow oxygen inhalation (Pattinson et al., 2005). Cai et al. reveals that oxygen enrichment can ameliorate high-altitude hypoxia-induced cognitive impairments associated with improved hippocampal morphology and molecular expression, and highlights that oxygen enrichment may become a promising alternative treatment against neurodegeneration for humans ascending to the plateau (Cai et al., 2021).

### 6.2 Medication

At present, drug therapy is also widely studied. The search for safe and effective treatment drugs has become one of the hot spots in the current plateau medical community. According to different action mechanisms, drugs are divided into acetylcholinesterase inhibitors, neurotrophic, hormone, antioxidant, traditional Chinese medicine and others.

#### 6.2.1 Acetylcholinesterase inhibitors (AChEIs)

Cholinergic system plays a great role in learning and memory research (Brinza et al., 2022). Several studies have shown that physostigmine (Muthuraju et al., 2009), galantamine (Muthuraju et al., 2009) and huperzine^24^ can restore the level of acetylcholine by blocking the activity of acetylcholinesterase, thus improving the cognitive impairment induced by HH. Furthermore, the AChEIs also reduce the AChE level in cortical and hippocampal neurons, which may prevent neurodegeneration (Muthuraju et al., 2009).

Another animal experiment in mice indicate that after the zinc chelator supplementation, the downregulation of AChE activity, choline acetyltransferase and muscarinic receptor 1 and 4 due to HH increased to different degrees. The study indicates that the zinc chelator supplementation might play important role in the neuronal damage and the alteration in cholinergic function associated with HH-induced memory impairment, and zinc chelation may be a promising treatment for HH-induced cognitive dysfunction (Udayabanu et al., 2012).

Shouzhangshen is a commonly used Tibetan drug in HAs in Western China. Several studies have revealed that shouzhangshen can enhance acetylcholinesterase expression, which may indicate a protective effect on cholinergic neurons (Zhang et al., 2006). Zhang et al. found that shouzhangshen extract had protective effect on nerve injury caused by acute high-altitude hypoxia in mice by reducing the expression levels of HIF-1α, VEGF, and MDA and by increasing SOD and GSH activity (Zhang et al., 2021).

#### 6.2.2 Antioxidants

The salidroside is a well-known medicinal plant with antioxidant potential (Zhang et al., 2013b). Zhang et al. indicated that salidroside could improve the memory acquisition and retrieval effectively of rat during HH, meanwhile, salidroside could also and increase mitochondrial biogenesis in rat brain (Barhwal et al., 2015). The possible mechanism was salidroside upregulation of phosphorylated cAMP response element binding protein through increased sirtuin one activity (Barhwal et al., 2015). This finding is consistent with that of Yang et al. who showed that salidroside could ameliorate the cognitive function of rats with HH by reducing the oxidative stress reaction in the hippocampus to alleviate the damage in the hippocampus (Yang et al., 2011).

Quercetin, which is widely exists in many plants, has been reported to prevent neuronal injury-induced ischemia (Zhang Z. A. et al., 2022). Liu et al. indicate that quercetin could promote a mitochondrial and neuron function adaptation through PGC-1alpha pathway, so as to improve the HH-induced memory injury (Liu et al., 2015). In addition, studies shows that quercetin reverses cognitive dysfunction by enhancing the antioxidant status during exposure to hypobaric hypoxia and improving the free radical scavenging enzyme system, reducing the ROS level in the hippocampus and subsequent lipid peroxidation (Prasad et al., 2013).

Epigallocatechin-3-gallate (EGCG) comes from green tea and has the following neuroprotective characteristics, such as metal chelation, suppression of oxidative stress and inflammation, and promotion of nerve regeneration (Cai et al., 2014). Chen et al. reports that EGCG could reduce cognitive dysfunction, iron deposition, oxidative stress and apoptosis, which were induced by HH. Meanwhile, EGCG could also promote neuronal regeneration against chronic high-altitude hypoxia-induced neural injury (Chen et al., 2022).

5,6,7,8-Tetrahydroxyflavone (THF), a flavone with four consecutive hydroxyl groups in ring A, exhibited effective antioxidant activity *in vitro* and *in vivo*. THF treatment could improve HH-induced cognitive dysfunction through suppressed oxidative stress. The mechanism is that THF could inhibit the levels of hydrogen peroxide and malondialdehyde, and increase the producing of glutathione and superoxide dismutase in brain tissue (Jing et al., 2022).

Verbascoside is a water-soluble natural phenylethanoid glycoside and presents widely in plants. It has been reported with multiple activities, such as antioxidant, neuroprotective activity, anti-inflammatory, anti-bacterial and immunomodulatory effect (Zhou et al., 2016). Li et al. indicated that treatment with verbascoside could reduce the working memory error and reference memory error significantly, it could also decrease the total errors and total time. These effects may be performed through relieving the neuron damage in CA1 region of the hippocampus and decreasing the activity of oxidative stress associated enzyme in plasma, brain and hippocampus. The mechanism research indicated that the improvement of HA-induced memory impairment mediated by verbascoside was associated with the regulation of oxidative stress and mTOR signaling pathway (Li et al., 2019).

Crocin is the main bioactive ingredients of Crocus sativus L., which has been reported to have avail on learning and memory (Khalili and Hamzeh, 2010). Zhang et al. found that crocin showed a cognitive protective effect by regulating SIRT1/PGC-1alpha pathways in rat’s hippocampus under hypoxia environment (Zhang X. Y. et al., 2018). Further study identified that the mechanism of crocin mediated cognitive protective was through accelerating mitochondrial biosynthesis, improving oxidative stress injury, and inhibiting neuronal apoptosis (Zhang X. et al., 2020).

Astragalus is one of the largest flowering plant genera of legumes, and its main components are Astragalus arepolysaccharides, flavonoids, Astragalosides (Wang et al., 2019). The extract of astragalus membranaceus can obviously ameliorate cognitive function in rats under hypoxia environment. The potential mechanism is involved in regulating oxidative stress, decreasing the accumulation of free radicals, and activating mTOR signaling pathway (Du et al., 2022).

Exposure to hypoxic conditions may induce the mitochondria-mediated apoptosis in hippocampal neurons, and consequently leading to cognitive dysfunction. Protecting mitochondrial function is a new method to treat cognitive dysfunction caused by hypobaric hypoxia. Hota et al. exposed SD rats to a hypobaric oxygen chamber with a simulated height of 7620m, and gave acetyl L-carnitine for 2 weeks, which could effectively protect hippocampal neurons from mitochondrial dysfunction, excitotoxicity and neurodegeneration (Hota et al., 2012). Further research showed that acetyl L-carnitine calcium buffering into nonfunctional mitochondria could improve excitotoxicity and bioenergetics status in hippocampal neurons (Hota et al., 2012).

#### 6.2.3 Neurotrophic

Calcium ion antagonists, such as nimodipine and iradipine, can selectively block Ca^2+^ channels on nerve cells and vascular endothelial cells. They not only promote learning and memory ability, but also alleviate nerve cell damage and improve memory dysfunction caused by hypoxia (Shao et al., 2020; Uema et al., 2021). In addition, iradipine can reduce the release of oxygen free radicals and cytochrome C caused by hypoxia, and antagonize the memory impairment caused by hippocampal CA1 neuron damage induced by HH(Barhwal et al., 2009).

Memantine is a non-competitive antagonist of glutamic acid NMDA receptors, widely used in the treatment of Alzheimer’s disease. The excitotoxicity mediated by glutamate through its receptor signals is considered to be one of the mechanisms of neuronal damage and cognitive dysfunction after exposure to HH. Current research has shown that chronic hypoxia can produce excitotoxicity, leading to nerve damage and cognitive dysfunction. Memantine treatment can suppress this toxicity by inhibiting excitotoxicity (Ji et al., 2021).

Propolis, a resinous substance produced by honey bees as a defense against intruders, has anti-oxidative and anti-inflammatory effects (Banskota et al., 2001). Zhu et al. demonstrate that propolis improves the cognitive function of elderly people living at HA by ameliorating microglial-mediated neuroinflammation and maintaining synaptic plasticity. Propolis could be used as alternative treatments for the cognitive decline in mild cognitive impairment and for reducing the risk of Alzheimer’s disease (Zhu et al., 2018).

Dl-3n-butylphthalide (NBP) extracted from celery has extensive neuroprotective effects. Min et al. suggest that NBP can improve the learning and memory ability of rats with chronic intermittent hypobaric hypoxia, because NBP increases the expression of brain-derived neurotrophic factor and promotes the phosphorylation of cAMP responsive element-binding, which is consistent with the results of Morris water maze (Min et al., 2014).

Echinacea glycoside (ECH) is a phenylethanol isolated from the stems of Cistanche deserticola, which has been reported to prevent ischemia traditionally caused by neuronal damage (Lu et al., 2018; Chen et al., 2019). Zheng et al. found that ECH can prevent HH-induced memory impairment through antioxidant activity in the hippocampus (Zheng et al., 2019). *In vivo*, ECH increases the expression of nuclear factor E2 p45 related factor 2, heme oxygenase-1and γ-glutamyl cysteine synthase, indicating that the Keap1-Nrf2-ARE signaling pathway may be involved in neuronal adaptation (Zheng et al., 2019).

Puerarin is the main bioactive component extracted from Pueraria lobata, which is known as Pueraria lobata in traditional Chinese medicine. Puerarin is a phytoestrogen that has various pharmacological effects, such as preventing and treating nervous system diseases. A recent study demonstrates that i. n. puerarin thermosensitive *in situ* hydrogels (TISGs) led to excellent brain targeting effect. Puerarin TISGs is an effective neuroprotective agent used to prevent brain damage caused by acute HH (Ma J. et al., 2020).

#### 6.2.4 Hormones

It has previously been reported that higher plasma cortisol levels can have profound effects on brain and advanced cognitive function after exposure to stress like ischemia (Faraji et al., 2009). Baitharu et al. indicated that the administration of corticosterone synthesis inhibitor to rats in the chronic hypobaric hypoxia environment could significantly improve the memory impairment induced by HA (Baitharu et al., 2012). The mechanism of action is related to increasing the ATP level in the hippocampus, regulating apoptosis markers, and reducing the expression of calcium channels.

Melatonin (MEL) is an endogenous neurohormone that has a variety of biological functions, including strong antioxidant effects (Carloni et al., 2008; Alonso-Alconada et al., 2013). Alonso et al. reported that melatonin is a neuroprotective antioxidant in both normoxic and hypobaric hypoxia, which can prevent and counteract the harmful effects of oxidative stress (reactive astrocyte proliferation, memory impairment, neuronal death and cognitive impairment) (Vornicescu et al., 2013). Melatonin supplementation may be a useful neuroprotective agent for the treatment of HH induced cognitive impairment.

Guanfacine belongs to the central nervous system α 2 Receptor agonists can improve prefrontal lobe disorder, neurodegenerative changes, and altitude induced spatial memory disorder. The regulation of guanfacine on adrenergic mechanism may play a role by improving prefrontal lobe defect and neurodegenerative changes during hypobaric hypoxia (Kauser et al., 2014).

Previous studies on the mechanism of HH induced cognitive dysfunction have mainly used small mammalian models such as mice and rats. Zhang et al. found that cynomolgus monkeys were fed progesterone (PROG) and steroid neuroprotective (TRIOL) can significantly alleviate cognitive dysfunction and salvage transcriptome changes induced by HH (Zhang P. et al., 2020). Functional studies of affected genes have shown that these two neuroprotective agents protect the brain through different targeted pathways, PROG enhances erythropoiesis, and TRIOL inhibits glutamate induced excitotoxicity (Zhang P. et al., 2020).

#### 6.2.5 Traditional Chinese medicine

Traditional Chinese medicine and Tibetan medicine are considered effective solutions for the prevention and treatment of cognitive dysfunction, and have been used for thousands of years in Asia. Currently, various evidences have shown that supplementing herbal medicines can regulate neurogenesis and protect hippocampal functions, such as memory (Iriti et al., 2010; Ye et al., 2016). Ganoderma lucidum has extensive therapeutic effects and neuroprotective effects on various diseases (Zhong et al., 2015; Huang et al., 2017). Sharma et al. found that in the Morris water maze test, Ganoderma lucidum can prevent hippocampal damage and spatial memory disorders caused by vascular brain edema in rats exposed to HH (Sharma and Tulsawani, 2020).

Cordycepin is the first nucleoside antibiotic isolated from fungi. It has biological activities such as lung and kidney protection, anti-tumor, neuroprotective, anti-inflammatory, antioxidant and immune regulation. Liu et al. indicate that cordycepin ameliorate HH-induced neuro inflammation, blood-brain barrier disruption, and cognitive dysfunction by inhibiting the TLR-4/NF-κB/MMP-9 pathway (Liu et al., 2022).

Monkey bread leaf extract, a known memory enhancer, can provide neuroprotection and improve memory impairment in HH (Stough et al., 2001). Hota et al. have demonstrated that bacoside can enhance the learning ability of rats, enhance memory recovery, and prevent dendritic atrophy after HH (Hota et al., 2009). In addition, bacoside also reduces plasma corticosterone levels, oxidative stress, and neuronal degeneration. Bacoside supplementation also increased the activity of cytochrome c oxidase, while ATP levels also increased. Therefore, administration of bacoxazole may be a useful therapeutic strategy in improving HH induced cognitive dysfunction and other related neurological diseases.

#### 6.2.6 Others

Acetazolamide is commonly used to treat acute mountain sickness. Some studies have confirmed that acetazolamide has a good effect in improving the oxygen tolerance and working ability of the body, but there are few studies on the impact of acetazolamide on cognitive ability in the hypoxic environment (White, 1984; Basnyat et al., 2008). However, a randomized controlled trial showed that after 3 days of HH exposure, compared with the placebo, acetazolamide not only has no protective effect on neuropsychological performance, but also causes more neuropsychological impairments (Wang et al., 2013). Therefore, it should be administered with caution.

## 7 Future direction for the neurocognitive function research at HH

To prevent and treat cognitive impairment caused by HH, comprehensive research is crucial. Firstly, we need to broaden the scope of research on cognitive function and conduct larger studies to examine the impact of altitude on all aspects of cognitive function. Secondly, we should intensify research efforts to uncover the mechanisms of brain lesions induced by high-altitude environments, identify the targets and mechanisms via which the brain adapts to HH exposure, and explore methods of adapting to and alleviating the effects of high-altitude environments. Thirdly, we should consider various factors such as length of stay at HA, exercise status, and mountain environment, and expand our research population to better understand the effects of altitude on individual mood changes. Fourthly, since most research on this topic is confined to animal models, conducting randomized controlled trials with a larger sample size is necessary to facilitate the clinical use of findings. Apart from that, we need to conduct more field research on HAs, since there are significant differences between simulated and actual high-altitude environments that can affect cognitive function in different ways. Finally, traditional Chinese medicine and Tibetan medicine, which have fewer side effects and are highly effective in preventing and treating cognitive impairment induced by HA, are great candidates for further research.

## 8 Conclusion

Long-term exposure to HH environment leads to cognitive dysfunction, which is manifested in features such as attention, learning, memory, and the processing ability. In this paper, the underlying molecular mechanism by which long-term HH exposure affects cognitive dysfunction is discussed. We also reviewed the progress in prevention and treatment of cognitive impairment caused by HH, and found that natural substances, such as traditional Chinese medicine and Tibetan medicine have good effects. However, these studies are limited to the animal experiment level. Future research needs to focus on human controlled trials and clinical trials to confirm their effectiveness.
